# Associations of parecoxib and other variables with recovery and safety outcomes in total knee arthroplasty: insights from a retrospective cohort study

**DOI:** 10.3389/fsurg.2023.1308221

**Published:** 2024-01-04

**Authors:** Ching-Yuan Hu, Jen-Hung Wang, Tsung-Ying Chen, Po-Kai Wang

**Affiliations:** ^1^ Department of Anesthesiology, Hualien Tzu Chi Hospital, Buddhist Tzu Chi Medical Foundation, Hualien, Taiwan; ^2^ School of Medicine, Tzu Chi University, Hualien, Taiwan; ^3^ Department of Medical Research, Hualien Tzu Chi Hospital, Buddhist Tzu Chi Medical Foundation, Hualien, Taiwan

**Keywords:** total knee arthroplasty, analgesia, parecoxib, mobilization, postoperative pain, rehabilitation

## Abstract

**Background:**

Early mobilization post-total knee arthroplasty (TKA) significantly affects patient outcomes. While parecoxib is known to reduce postoperative pain and morphine use with a favorable safety profile, its impact on mobilization timing post-TKA remains uncertain. This retrospective study aims to assess parecoxib's influence on postoperative mobilization timing in TKA patients without compromising safety.

**Methods:**

This study included unilateral TKA patients treated for primary knee osteoarthritis under general anesthesia. We divided the study period into two intervals, 2007–2012 and 2013–2018, to evaluate temporal differences. Both the control group and parecoxib group received standard postoperative oral analgesics and as-needed intramuscular morphine. The control group did not receive parecoxib, while the parecoxib group did. Primary outcomes compared postoperative complications and mobilization timing between groups, with secondary outcomes including length of hospital stay (LOS), Visual Analog Scale (VAS) scores for pain, as-needed morphine use, and postoperative nausea/vomiting.

**Results:**

Parecoxib did not increase postoperative complications. Unmatched comparison with patients in controlled group found that patients in parecoxib group had significantly shortened mobilization time (2.2 ± 1.1 vs. 2.7 ± 1.6 days, *P* < 0.001) and LOS (6.7 ± 2.5 vs. 7.2 ± 2.1 days, *P* = 0.01). Multivariate analysis linked parecoxib use with faster mobilization (*β *= −0.365, *P* < 0.001) but not LOS. Males showed increased mobilization time and LOS compared to females during the period of 2007–2018, but gender had no significant association with LOS during the period of 2013–2018. The 2013–2018 period saw significant reductions in both mobilization time and LOS. Use of a tourniquet and local infiltration analgesia showed no significant impact. ASA classification 1–2 was positively associated with faster mobilization but not LOS. Longer operation times were linked to delayed mobilization and increased LOS.

**Conclusion:**

In this study, intravenous parecoxib injection, female gender, and shorter OP time had consistent positive association with shorter time to mobilization after individual multivariate analysis in 2 different period. The use of parecoxib had consistent no significant association with LOS. Only shorter OP time was consistent positive associated with shorter LOS.

## 1 Introduction

Total knee arthroplasty (TKA) stands as a prevalent surgical intervention, serving as an effective treatment modality for advanced knee osteoarthritis. The postoperative rehabilitation process is critically linked to patients' functional recovery and influences the timing of hospital discharge following TKA (1, 2). Early mobilization within the rehabilitation regimen serves as an essential factor for enhanced knee functionality. Studies indicate that initiating mobilization as early as within a week post-TKA contributes to lower D-dimer levels and numerous other benefits, including shortened length of hospital stay (LOS), cost-effectiveness, improved knee functionality, and reduced incidence of deep vein thrombosis (DVT) and pulmonary infections (3–7).

Patients undergoing TKA typically experience moderate to severe postoperative pain (8, 9). Effective pain management not only alleviates postoperative discomfort but potentially offers additional benefits, such as reduced bed rest duration and accelerated functional recovery (10). Current protocols advocate for multimodal analgesia, emphasizing opioid-sparing strategies to mitigate opioid-associated adverse effects. Consequently, non-steroidal anti-inflammatory drugs (NSAIDs) have become the preferred postoperative analgesic agents globally, owing to their efficacy in pain relief and fewer opioid-related adverse events, such as nausea and vomiting (11–16).

Parecoxib sodium, a selective cyclooxygenase-2 (COX-2) inhibitor and the parenteral prodrug of valdecoxib, has been established as a safe alternative for postoperative pain management (17–20). Existing literature confirms its efficacy in alleviating pain, reducing opioid consumption, and minimizing opioid-related adverse effects in diverse surgical settings, including TKA (21–24).

Despite these advancements, it remains unclear whether the use of parecoxib exerts an influence on rehabilitation timelines and LOS in TKA patients. Moreover, additional variables that may modulate patient responses to postoperative pain and recovery while using parecoxib have not been adequately elucidated.

In this retrospective cohort study, the aim is to investigate the potential influence of parecoxib on the timing of postoperative mobilization in patients undergoing TKA, without compromising safety.

## 2 Materials and methods

### 2.1 Ethical approval and study design

This retrospective case-control study received approval from the Institutional Review Board of Hualien Tzu Chi General Hospital (Ethics Committee Number: IRB106-69-B). We reviewed electronic medical records for patients who underwent unilateral primary TKA for primary knee osteoarthritis between January 1, 2007, and April 30, 2018. The enrolled period was more than 11 years and the time zone was divided into two parts, 2007–2012 and 2013–2018, to compare whether there are differences between the two groups before and after the time zone change.

### 2.2 Study objectives

The primary objectives were to evaluate whether parecoxib administration accelerates time to mobilization post-TKA, and investigated postoperative complications possibly related to parecoxib administration, including in-hospital death, myocardial infarction, stroke, upper gastrointestinal (GI) bleeding, unpredictable ICU care, and blood transfusion. Secondary objectives included comparative analysis between parecoxib and control groups in terms of LOS, postoperative visual analogue scale (VAS) scores (0 represented no pain and 10 represented the worst pain), morphine consumption, and identification of variables potentially influencing study outcomes.

The definition of time to mobilization is measured in days, starting from the day of surgery as Day Zero. It records the number of days until the patient is able to get out of bed and walk using a four-legged walker for a short distance. Since all patients were admitted to the hospital the day before their surgery, the LOS is calculated starting from the day before surgery, including the day of discharge as a full day. Morphine consumption was determined by calculating the dosage of intramuscular as-needed opioid injection over the first 3 days post-surgery. While the majority of the as-needed opioids were morphine-based, we have converted the dosages of various postoperative opioids into their morphine equivalent units.

### 2.3 Inclusion and exclusion criteria

The choice of anesthetic plan is determined by anesthesiologists after evaluating the patients' physical status. Patients were eligible if they underwent general anesthesia managed with a laryngeal mask airway (LMA) and were scheduled for TKA. Endotracheal general anesthesia (ETGA) is used for patients with special conditions, including a BMI over 40, a high risk of aspiration pneumonia, liver cirrhosis with ascites, and intraoperative ventilation issues. Patients receiving ETGA were excluded from both groups to reduce possible research bias. Exclusion criteria also included patients who underwent neuraxial anesthesia, or regional anesthesia. Besides, we excluded patients who received postoperative pain management via nerve block, intravenous or epidural patient-controlled analgesia, or intrathecal analgesia.

### 2.4 Group allocation

Patients were categorized into two groups: the control group and the parecoxib group. The control group consisted of patients who received regular oral pain medication but did not receive intravenous (IV) parecoxib. The parecoxib group comprised patients who received a single IV dose of parecoxib prior to anesthetic induction and continued with the administration postoperatively for at least 1 day or longer.

### 2.5 Anesthetic plan and postoperative pain management

All procedures were conducted under general anesthesia facilitated by LMA. Anesthetic induction included fentanyl (50 µg), lidocaine (30–40 mg), and propofol (1–2 mg/kg). Anesthesia was maintained using sevoflurane, adjusted to MAC levels of 1.0–1.3, and supplemented with IV fentanyl (25 µg) as required. The perioperative dosage of fentanyl and the use of tourniquet for surgical facilitation would be recorded to conduct data analysis. For patients who received local infiltration analgesia (LIA) during the operation (OP), we also gathered their records and conducted data analysis. The agents of LIA included bupivacaine, ketorolac, tranexamic acid, and epinephrine.

Pain management encompassed both pharmacological and non-pharmacological strategies. The pharmacological regimen included a combination of oral tramadol and acetaminophen for at least 3 days. Parenteral morphine was administered as needed for moderate to severe pain, 4–6 h postoperatively. The parecoxib regimen involved administering two to six intravenous injections of 40 mg each during the first 3 days after surgery, which included an initial single dose given intravenously before the induction of anesthesia.

### 2.6 Non-pharmacological interventions

Non-pharmacological pain management, including cold application, muscle relaxation, and massage, was the same in both study groups.

### 2.7 Discharge criteria

The general discharge criteria for all patients who have undergone TKA typically include several key factors that ensure the patient's safety and readiness for home recovery:

1. Patients should be able to manage their pain with oral medication and without the need for parenteral pain relief.
2. Patients must demonstrate the ability to walk a certain distance (often with the aid of a walker or crutches) and perform basic movements without significant assistance.
3. Achieving a specified range of motion in the knee joint (at least 90 degrees of flexion) is a criterion.
4. The patient's vital signs should be stable, and there should be no signs of significant medical complications such as infection, excessive bleeding, or unstable cardiovascular status.
5. Patients should be able to perform basic self-care activities and have a plan for continued rehabilitation and care at home.

### 2.8 Data collection and analysis

We compiled patient demographics and clinical characteristics, including age, gender, body mass index (BMI), hypertension status, diabetes mellitus, ASA physical status, and OP time. Outcomes were analyzed for both primary and secondary objectives.

### 2.9 Statistical analysis

Continuous variables were compared using either independent *t*-tests or Wilcoxon rank-sum tests, while categorical variables were analyzed using Chi-squared or Fisher's exact tests. For the assessment of effect sizes across studies, standardized mean difference (SMD) was employed. Variables are presented as mean (SD), count (%), and range where applicable. Additionally, a multiple linear regression model was utilized to assess the association between potential influencing variables and study outcomes, with SMD values providing insights into effect sizes. Statistical significance was set at *P* < 0.05. All analyses were performed using SPSS version 17.0 (SPSS Inc., Chicago, IL, USA).

## 3 Results

### 3.1 Patient demographics and characteristics

**Figure 1:**
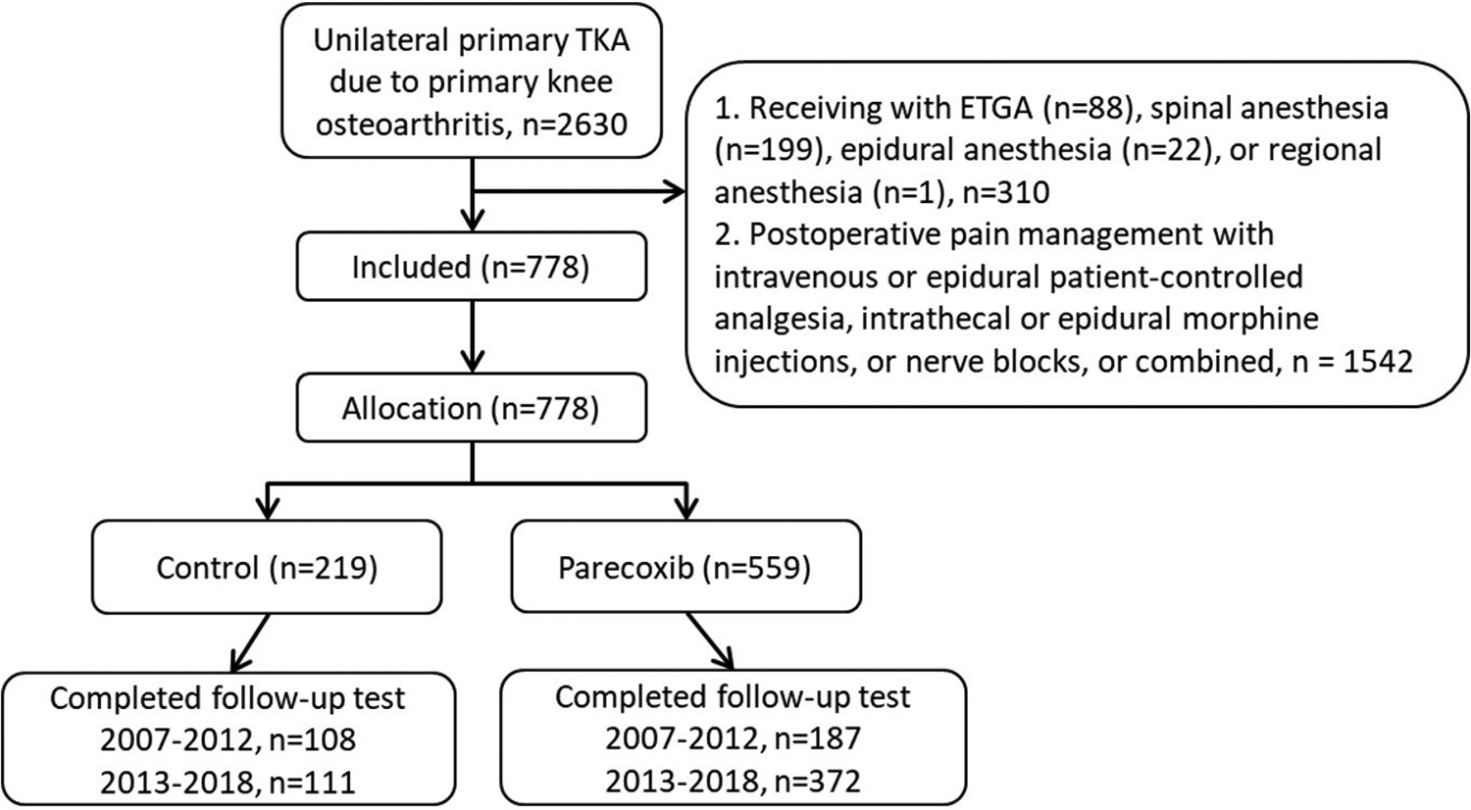
The flowchart of patient selection and allocation.

A total of 2,630 patients undergoing unilateral primary total knee arthroplasty (TKA) due to primary knee osteoarthritis were initially considered for this study (Figure 1). Of these, 310 patients were excluded due to the administration of general anesthesia via endotracheal tube intubation, neuraxial anesthesia, or regional anesthesia. Another 1,542 were excluded based on their postoperative pain management protocols, which included IV or epidural patient-controlled analgesia (PCA), neuraxial morphine injections, nerve blocks, or combined. Ultimately, 778 patients were enrolled, allocated to study groups, and completed follow-up. The control group comprised 219 patients, while the parecoxib group included 559 patients.

### 3.2 Baseline characteristics

Baseline characteristics of patients in both groups are summarized in Table 1. Patients in the parecoxib group were significantly older (68.9 ± 8.7 years) than those in the control group (67.2 ± 10.2 years) (*P* = 0.026). The parecoxib group had a significantly lower percentage of male patients (22.4%) and a higher percentage of female patients (77.6%) compared to the control group (male 34.7%, female 65.3%) (*P* < 0.001). A higher percentage of procedures in the parecoxib group were performed in the later period (2013–2018, 66.5%) as opposed to the earlier period (2007–2012, 33.5%). This was reflective of the overall trend, with 62.1% of all surgeries occurring in the later period (*P* < 0.001). The use of intraoperative tourniquets and LIA was comparable between the two groups, with no significant differences observed. The mean dosage of perioperative fentanyl was 0.12 mg in both groups, indicating uniformity in analgesic administration. Hypertension and diabetes mellitus were prevalent in 62.0% and 23.5% of all patients, respectively, with no significant differences between the control and parecoxib groups. The American Society of Anesthesiologists (ASA) Classification was similar between the groups, with the majority of patients falling into the 1–2 category (64.0%). Mean values of BMI were 28.1 kg/m^2^ in control group and 28.5 kg/m^2^ in parecoxib group with no significant difference between the groups. The operative time was significantly shorter in the parecoxib group (110.6 ± 24.0 min) than in the control group (121.9 ± 29.1 min) (*P* < 0.001). The SMD values for these characteristics ranged from 0.01 to −0.42, providing a measure of effect size and variance between the groups. Baseline characteristics of patients in both groups during the period of 2013–2018 are summarized in Supplementary Table S1, and the results of analysis was similar as Table 1, including older patients, less percentage of male, and shorter OP time in parecoxib group compared to the control group.

**Table 1:** Patient demographics (*N* = 778).

	Control	Parecoxib	Total	*P*-value	SMD
*N*	219	559	778		
Mean age (SD) in years	67.2 (10.2)	68.9 (8.7)	68.4 (9.2)	0.026*	0.17
Gender, *n* (%)				0.001*	−0.28
Male	76 (34.7)	125 (22.4)	201 (25.8)		
Female	143 (65.3)	434 (77.6)	577 (74.2)		
Period, *n* (%)				<0.001*	−0.33
2007–2012	108 (49.3%)	187 (33.5%)	295 (37.9%)		
2013–2018	111 (50.7%)	372 (66.5%)	483 (62.1%)		
Intraoperative tourniquet use, *n* (%)	213 (97.3%)	546 (97.7%)	759 (97.6%)	0.736	0.03
LIA, *n* (%)	10 (4.6%)	36 (6.4%)	46 (5.9%)	0.332	0.08
Mean dosage of perioperative fentanyl (SD) in mg	0.12 (0.08)	0.12 (0.08)	0.12 (0.08)	0.684	0.03
Hypertension, *n* (%)	134 (61.2)	348 (62.3)	482 (62.0)	0.783	0.02
Diabetes mellitus, *n* (%)	52 (23.7)	131 (23.4)	183 (23.5)	0.927	−0.01
ASA classification, *n* (%)				0.526	−0.05
1–2	144 (65.8)	354 (63.3)	498 (64.0)		
≧3	75 (34.2)	205 (36.7)	280 (36.0)		
Mean BMI (SD) in kg/m^2^	28.1 (4.7)	28.5 (5.0)	28.4 (4.9)	0.213	0.1
Mean OP time (SD) in minutes	121.9 (29.1)	110.6 (24.0)	113.8 (26.0)	<0.001*	−0.42

SMD, standardized mean difference; SD, standard deviation; LIA, local infiltration analgesia; ASA, American society of anesthesiologists; BMI, body mass index; OP, operation.

Data are presented as *n* (%) or mean (SD).

* *P*-value < 0.05 was considered statistically significant after test.

### 3.3 Postoperative complications

Table 2 details postoperative complications, including in-hospital mortality, myocardial infarction, stroke, upper gastrointestinal (GI) bleeding, unplanned ICU admission, and blood transfusion. No significant differences were observed between the two groups in these variables. The SMD values for these characteristics ranged from 0 to −0.14, providing a measure of effect size and variance between the groups. During the period of 2013–2018, no significant differences were also observed between the two groups in these variables (Supplementary Table S2).

**Table 2:** Postoperative complications (*N* = 778).

	Control	Parecoxib	Total	*P*-value	SMD
*N*	219	559	778		
In-hospital death, *n* (%)	0 (0.0)	0 (0.0)	0 (0.0)	1	0
Myocardial infarction, *n* (%)	0 (0.0)	0 (0.0)	0 (0.0)	1	0
Stroke, *n* (%)	0 (0.0)	0 (0.0)	0 (0.0)	1	0
Upper GI bleeding, *n* (%)	2 (0.9)	0 (0.0)	2 (0.3)	0.079	−0.14
Unpredictable ICU care, *n* (%)	1 (0.5)	1 (0.2)	2 (0.3)	0.484	−0.05
Blood transfusion, *n* (%)	44 (20.1)	82 (14.7)	126 (16.2)	0.065	−0.14

SMD, standardized mean difference; NSAIDs, non-steroidal anti-inflammatory drugs; GI, gastrointestinal; ICU, intensive care unit.

Data are presented as *n* (%).

### 3.4 Primary and secondary outcomes

Table 3 shows that the time to mobilization post-surgery was significantly shorter in the parecoxib group (2.2 ± 1.1 days) compared to the control group (2.7 ± 1.6 days) (*P* < 0.001). LOS was also shorter in the parecoxib group (6.7 ± 2.5 days) than in the control group (7.2 ± 2.1 days) (*P* = 0.010). The VAS score on postoperative day 1 was significantly lower in the parecoxib group (3.1 ± 1.2) compared to the control group (3.3 ± 1.5) (*P* = 0.028). There were no statistically significant differences in VAS scores on postoperative days 2 and 3. The total morphine dose in the first three postoperative days was significantly lower in the parecoxib group (8.4 ± 6.9 mg) than in the control group (16.4 ± 13.9 mg) (*P* < 0.001). There were no statistically significant differences in postoperative nausea and vomiting. The SMD values for these characteristics ranged from −0.02 to −0.71, providing a measure of effect size and variance between the groups. During the period of 2013–2018 (Supplementary Table S3), the time to mobilization post-surgery was significantly shorter in the parecoxib group (2.0 ± 0.9 days) compared to the control group (2.4 ± 1.4 days) (*P* = 0.002). LOS was not significantly different between the parecoxib group (6.5 ± 2.4 days) than in the control group (6.8 ± 2.0 days) (*P* = 0.167). There were no statistically significant differences in VAS scores in first three postoperative days. The total morphine dose in the first three postoperative days was significantly lower in the parecoxib group (9.0 ± 7.2 mg) than in the control group (14.4 ± 11.4 mg) (*P* < 0.001). There were no statistically significant differences in postoperative nausea and vomiting.

**Table 3:** Primary and secondary outcomes (*N* = 778).

	Control	Parecoxib	Total	*P*-value	SMD
*N*	219	559	778		
Primary outcome					
Time to mobilization (SD) in days	2.7 (1.6)	2.2 (1.1)	2.4 (1.2)	<0.001*	−0.35
Secondary outcomes					
Mean LOS (SD) in days	7.2 (2.1)	6.7 (2.5)	6.9 (2.4)	0.010*	−0.21
Postoperative VAS (SD)					
Day 1	3.3 (1.5)	3.1 (1.2)	3.2 (1.3)	0.028*	−0.17
Day 2	2.8 (1.1)	2.8 (1.0)	2.8 (1.0)	0.789	−0.02
Day 3	2.7 (0.9)	2.6 (1.0)	2.6 (1.0)	0.198	−0.1
Mean dosage of morphine (SD) in mg					
Day 1	8.9 (6.0)	6.5 (4.5)	7.2 (5.1)	<0.001*	−0.42
Days 1–2	14.4 (11.5)	7.9 (6.1)	9.7 (8.5)	<0.001*	−0.66
Days 1–3	16.4 (13.9)	8.4 (6.9)	10.6 (10.1)	<0.001*	−0.71
Postoperative nausea and vomiting, *n* (%)	34 (15.5%)	80 (14.3%)	114 (14.7%)	0.667	−0.03

SMD, standardized mean difference; SD, standard deviation; VAS, visual analogue scale; LOS, length of stay.

Data are presented as mean (SD).

* *P*-value < 0.05 was considered statistically significant after test.

### 3.5 Multivariable analyses

To evaluate the influence of variables other than parecoxib on study outcomes, multiple linear regression models were utilized. The results for each variable are presented in Tables 4, 5.

**Table 4:** Variables associated with time to mobilization (days) (*N* = 778).

Predictor	Multivariate	
	*β* (95% CI)	*P*-value
Group (parecoxib vs. control)	−0.365 (−0.563, −0.167)	<0.001*
Age	−0.010 (−0.021, 0.001)	0.074
Gender (male vs. female)	0.259 (0.050, 0.469)	0.015*
Period (2013–2018 vs. 2007–2012)	−0.616 (−0.798, −0.435)	<0.001*
Tourniquet (yes vs. no)	0.400 (−0.147, 0.947)	0.152
LIA (yes vs. no)	0.192 (−0.173, 0.557)	0.303
Hypertension (yes vs. no)	−0.029 (−0.220, 0.162)	0.767
Diabetes mellitus (yes vs. no)	0.003 (−0.209, 0.214)	0.98
ASA classification (1–2 vs. ≧3)	−0.281 (−0.476, −0.086)	0.005*
OP time	0.005 (0.002, 0.009)	0.005*

LIA, local infiltration analgesia; ASA, American society of anesthesiologists; OP, operation; CI, confidence interval.

Dependent variable: postoperative time to mobilization.

* *P*-value < 0.05 was considered statistically significant after test.

**Table 5:** Variables associated with length of stay (LOS) (*N* = 778).

Predictor	Multivariate	
	*β* (95% CI)	*P*-value
Group (parecoxib vs. control)	−0.178 (−0.552, 0.196)	0.35
Age	−0.017 (−0.037, 0.002)	0.081
Gender (male vs. female)	0.509 (0.118, 0.901)	0.011*
Period (2013–2018 vs. 2007–2012)	−0.638 (−0.990, −0.286)	<0.001*
Tourniquet (yes vs. no)	0.171 (−0.903, 1.245)	0.755
LIA (yes vs. no)	−0.018 (−0.727, 0.691)	0.96
Hypertension (yes vs. no)	0.312 (−0.049, 0.674)	0.091
Diabetes mellitus (yes vs. no)	0.037 (−0.364, 0.439)	0.855
ASA classification (1–2 vs. ≧3)	−0.208 (−0.574, 0.158)	0.266
OP time	0.018 (0.012, 0.025)	<0.001*

LIA, local infiltration analgesia; ASA, American society of anesthesiologists; OP, operation; CI, confidence interval.

Dependent variable: length of stay.

* *P*-value < 0.05 was considered statistically significant after test.

In Table 4, several variables were evaluated for their influence on time to mobilization. The parecoxib group showed a significantly shorter time to mobilization compared to the control group (*β* = −0.365, 95% CI: −0.563, −0.167, *P* < 0.001). Female patients also mobilized sooner than male patients, with a positive association observed (*β* = 0.259, 95% CI: 0.050, 0.469, *P* = 0.015). Comparing the years 2013–2018 with 2007–2012, and a significant reduction in time to mobilization was noted in the more recent period (*β* = −0.616, 95% CI: −0.798, −0.435, *P* < 0.001). The use of a tourniquet and LIA neither was found to be significantly associated with mobilization time in this study (Tourniquet: *β* = 0.400, 95% CI: −0.147, 0.947, *P* = 0.152; LIA: *β* = 0.192, 95% CI: −0.173, 0.557, *P* = 0.303). Patients with ASA classification 1–2 showed a beneficial effect on time to mobilization compared to those with ASA classification ≥3 (*β* = −0.281, 95% CI: −0.476, −0.086, *P* = 0.005).OP time had a significantly positive impact on time to mobilization (*β* = 0.005, 95% CI: 0.002, 0.009, *P* = 0.005). Conversely, age, hypertension, and diabetes mellitus demonstrated no significant association with time to mobilization. In Supplementary Table S4, parecoxib group, older age, female gender, and shorter OP time were the variables associated with shorter time to mobilization during the period of 2013–2018. Other variables demonstrated no significant association with time to mobilization.

In Table 5, several variables were analyzed for their association with LOS. The parecoxib group showed no significant association compared to the control group (*β* = −0.178, 95% CI: −0.552, 0.196, *P* = 0.35). Males exhibited a significant increase in LOS compared to females (*β* = 0.509, 95% CI: 0.118, 0.901, *P* = 0.011). The period of surgery had a substantial impact, with the 2013–2018 cohort exhibiting a significant decrease in LOS compared to the 2007–2012 cohort (*β* = −0.638, 95% CI: −0.990, −0.286, *P* < 0.001). No significant association with LOS was observed for the use of a tourniquet (*β* = 0.171, 95% CI: −0.903, 1.245, *P* = 0.755) or LIA (*β* = −0.018, 95% CI: −0.727, 0.691, *P* = 0.96). OP time also had a significantly positive association with LOS (*β* = 0.018, 95% CI: 0.012, 0.025, *P* < 0.001). Conversely, age, hypertension, diabetes mellitus, and ASA classification did not exhibit a significant relationship with LOS. In Supplementary Table S5, OP time had a significantly positive association with LOS (*β* = 0.019, 95% CI: 0.010, 0.028, *P* < 0.001) during the period of 2013–2018, and other variables demonstrated no significant association with LOS.

## 4 Discussion

In this retrospective study, we evaluated the impact of perioperative parecoxib on various postoperative outcomes such as complications, time to mobilization, length of stay (LOS), Visual Analog Scale (VAS) scores, and cumulative morphine dosage after total knee arthroplasty (TKA). There was a statistically significant improvement in the time to mobilization and LOS for patients who received parecoxib, with shorter average durations compared to the control group in Table 3. However, after multiple regression analysis, our study is pioneering in demonstrating a correlation between parecoxib administration and shortened time to mobilization post-TKA without compromising patient safety, but subsequent multivariate analysis indicated no significant correlation between parecoxib administration and LOS. Besides, shorter mobilization time was also associated with female gender, later period (2013–2018), ASA classification 1–2, and shorter OP time compared to the control group. Variables, including female gender, later period (2013–2018) and shorter OP time were related to shorter LOS. Neither the use of a tourniquet nor LIA within the perioperative setting significantly affects the time to mobilization and LOS. During the period of 2013–2018, shorted OP time was the only contributing factor to shorter LOS.

In line with a previous meta-analysis, intravenous parecoxib effectively mitigated postoperative knee pain and morphine requirements after TKA (25). Despite heterogeneity in the effects of parecoxib on VAS scores across studies, our data corroborated a reduction in postoperative pain and morphine use. Morphine dosage varied by gender and OP time, which may be attributed to differences in pain perception and analgesic response between sexes (25). Reduced OP time also correlated with decreased levels of pro-inflammatory markers like IL-6 and C-reactive protein, potentially requiring less morphine for pain management (26).

In Table 1, different patient demographics are characteristic of retrospective studies and such variations can occur naturally (27). We have ensured that these differences are accounted for in our analyses. Specifically, we employed statistical methods like regression to adjust for these baseline variations. This approach helps us to maintain the integrity of our findings, ensuring that the observed outcomes are not artifacts of baseline disparities but genuine effects of the interventions studied. We also conducted SMD statistical analysis for each demographic item in Table 1 to determine if there are significant differences. SMDs of 0.2, 0.5, and 0.8 are considered small, medium, and large respectively, and could be used in retrospective study (28). In Table 1, although patients in the parecoxib group were significantly older than those in the control group with less than small SMD (0.17), the age did not exhibit a significant relationship with time to mobilization and LOS. Gender, period, and OP time exhibited statistically significant results with small to medium SMD between two groups. After multiple regression analysis, gender, period, and OP time were the variables associated with time to mobilization and LOS, and the results implicated this study was adequately powered.

The variation in operative time, as highlighted, could be influenced by the later surgery period (2013–2018) in the Parecoxib group. The OP time in surgeries, such as those evaluated in our study, can be influenced by various factors beyond medication such as parecoxib. While parecoxib is known for its efficacy in reducing postoperative pain, its direct impact on OP time is less clear and not supported by the data in our study. Our results are in agreement with several other studies. In the Bohl's study, 165,474 patients were identified between 2006 and 2013 in the American College of Surgeons National Surgical Quality Improvement Program (NSQIP) database who underwent primary total hip or knee arthroplasty and an increase in operative time by 15 min increased the risk for LOS (≥4 days) by 9% (29). In the Sodhi's research, the NSQIP database was used to identify and obtain data for 225,344 primary TKA cases between 2008 and 2016 and OP times, analyzed as 30-min time intervals and as a continuous variable, were found to have significant associations with LOS (30). Factors that may lead to variations in OP times include patient characteristics, resident involvement, surgical technique and complexity, proficiency and technique of the surgeon, unforeseen complications during surgery, availability and efficiency of operating room resources (31–35). These factors highlight the multifaceted nature of OP time in surgical procedures. It's important to consider these aspects in the interpretation of our study results and in future research endeavors.

In this study, parecoxib was observed to facilitate early mobilization postoperatively, and several factors could be contributing to this phenomenon. Firstly, parecoxib has a strong analgesic effect, reducing the necessity for opioid medications, which are known to cause side effects that can hamper early mobilization and rehabilitation, such as sedation and constipation (36, 37). Secondly, the medication seems to influence pain threshold and inflammatory factors, thus aiding in quicker recovery and mobility. Lastly, parecoxib does not have a significant adverse effect on bone healing, which is a crucial aspect of post-operative recovery for orthopedic surgeries that heavily rely on early mobilization (38, 39). Therefore, parecoxib appears to create an environment conducive for early patient mobilization by acting on multiple fronts: analgesia, inflammation control, and negligible impact on bone healing.

This study found longer LOS for males compared to females but gender had no significant association with LOS during the period of 2013–2018. However, in several previous studies, female sex was a significant predictor of increased LOS (40, 41). Past research identified female gender as a risk factor for extended LOS, attributed to a higher likelihood of urinary tract infections and a marginal increase in venous thromboembolic events (42, 43). The gender difference in this study was not similar as the previous studies and there may have several possible reasons. First, the sample sizes were different (*N* = 483 in Supplementary Table S5 vs. *N* = 778 in Table 5). A larger sample size could provide a more precise estimate and may lead to the detection of statistically significant differences that smaller studies cannot. The periods of the studies were different, with Table 5 including data from an earlier period (2007–2012) compared to Supplementary Table S5 (2013–2018). Changes in medical practices or patient demographics over time could influence the results. A higher number of females receiving parecoxib might have influenced the overall faster LOS observed in the female subgroup, but it should be interpreted with caution while the data supports the observation. The presence of other confounding variables, which were limitations in this study, not accounted for in the analysis could also affect the results. The difference of results between this study and others may need further analysis and study to identify the possible factors or etiologies.

The later period (2013–2018) is associated with a shorter time for mobilization and length of stay (LOS) could be due to several factors. First, emphasizing rapid mobilization with standardized order sets and pathways is as a key component of recovery in patients undergoing TKA. Studies have shown that early mobilization is linked to improved outcomes and reduced LOS (4, 44). Over the years, there may have been improvements in surgical techniques, anesthesia, and pain management (45). Improved preoperative patient education about the importance of early mobilization and setting expectations for recovery may also contribute to these improved outcomes (45).

ASA classification also appeared to influence LOS; each increase by two ASA levels corresponded to an extended LOS by half a day (46). However, the present study did not find a significant association between ASA class and prolonged LOS. Two factors, clinical decision variability and overlapping factors, could potentially attenuate the impact of ASA classification on the LOS (47, 48). The assignment of an ASA Physical Status classification is inherently a clinical decision that involves multiple considerations. Many other important variables, such as surgical severity and the expertise of the medical team, are not encapsulated within the ASA classification. Therefore, its influence on LOS could be overshadowed by other, more immediate clinical factors.

The inclusion criteria and treatment modalities, including the use of parecoxib, were based on past clinical decisions and patient preferences, not predetermined study protocols. This approach reflects the real-world scenario where parecoxib use is contingent upon individual patient factors, including their willingness and any contraindications to the medication. It's important to note that our study did not exclude all patients who had contraindications to parecoxib. This inclusion could mean that some patients in the control group had more severe or different health conditions compared to the parecoxib group, including but not limited to cardiovascular disorders, peptic ulcers, or chronic kidney diseases. These health disparities might have led to a slower recovery in the control group, potentially introducing a bias in the results regarding the effect of parecoxib on postoperative mobilization after TKA. The inclusion of patients with contraindications to parecoxib in the control group, and the subsequent potential health disparities, is considered as a limitation of our study.

TKA can be performed using various surgical methods. These methods primarily differ in the type of surgical approach used to access the knee joint. In this study, we did not include the data of the different approaches of TKA and may have introduced some error in the results.

Although the ASA classification represents a patient's physiological status, and we have used it for analysis, we have obtained too few patient disease categories to further explore the relationship between disease categories and time to mobilization as well as length of stay (LOS). Perhaps this will be addressed in future research.

Besides, other limitations exhibit in this study. The number of patients between the two groups was unequal (219 in control group and 559 in parecoxib group), and the patients were neither randomized nor blinded. Although several variables were analyzed in this study, the study of retrospective cohort data is influenced by several factors, including the selection of an inappropriate outcome variable, the presence of sparse-data bias, the occurrence of collinearity among covariates, and the impact of comorbidities (49). Therefore, these factors warrant further prospective studies to validate our findings.

## 5 Conclusion

In this study, patients who underwent TKA with general anesthesia using a LMA and received intravenous parecoxib, female gender, and shorter OP time showed a consistently positive association with a shorter time to mobilization. This association was observed across two distinct periods after adjusting for individual multiple variables. However, the administration of parecoxib did not have a significant impact on the LOS in the hospital. The only factor that was consistently positively associated with a shorter LOS was a reduced operation time.

## Data Availability

The original contributions presented in the study are included in the article/**Supplementary Material**, further inquiries can be directed to the corresponding author.
