# Supplementary material for: Associations of parecoxib and other variables with recovery and safety outcomes in total knee arthroplasty: insights from a retrospective cohort study
Source: Front Surg. 2024 Jan 4;10:1308221. doi: 10.3389/fsurg.2023.1308221 (PMC10794493; doi:10.3389/fsurg.2023.1308221)
Supplement: Supplementary file 1 [file Datasheet1.pdf]

Supplement Data

Table S1. Patient Demographics During the Period of 2013-2018 (N=483)

	Control	Parecoxib	Total	P-value	SMD
N	111	372	483		
**Mean age (SD) in years**	66.7±10.0	68.9±8.4	68.4±8.8	0.025*	0.23
**Gender, n (%)**				0.016*	-0.25
Male	38(34.2%)	85(22.8%)	123(25.5%)		
Female	73(65.8%)	287(77.2%)	360(74.5%)		
**Intraoperative Tourniquet Use, n (%)**	105(94.6%)	359(96.5%)	464(96.1%)	0.403	0.09
**LIA, n (%)**	10(9.1%)	36(9.7%)	46(9.5%)	0.854	0.02
**Mean dosage of Perioperative Fentanyl (SD) in mg**	0.12±0.09	0.12±0.08	0.12±0.08	0.966	0.00
**Hypertension, n (%)**	64(57.7%)	230(61.8%)	294(60.9%)	0.429	0.09
**Diabetes mellitus, n (%)**	28(25.2%)	89(23.9%)	117(24.2%)	0.779	-0.03
**ASA Classification, n (%)**				0.063	-0.21
1~2	81(73.0%)	236(63.4%)	317(65.6%)		
≧3	30(27.0%)	136(36.6%)	166(34.4%)		
**Mean BMI (SD) in kg/m^2**	28.6±5.0	28.7±5.1	28.7±5.1	0.940	0.01
**Mean OP Time (SD) in minutes**	118.1±28.4	109.4±23.6	111.4±25.0	0.001*	-0.33

Data are presented as n (%) or mean (SD). *P-value < 0.05 was considered statistically significant after test.

SMD: Standardized mean difference, SD: standard deviation, LIA: local infiltration analgesia, ASA: American Society of Anesthesiologists, BMI: body mass index, OP: operation.

Table S2. Postoperative Complications During the Period of 2013-2018 (N=483)

	Control	Parecoxib	Total	P-value	SMD
N	111	372	483		
**In-hospital death, n (%)**	0(0.0%)	0(0.0%)	0(0.0%)	1.000	0.00
**Myocardial infarction, n (%)**	0 (0.0)	0 (0.0)	0 (0.0)	1	0
**Stroke, n (%)**	0 (0.0)	0 (0.0)	0 (0.0)	1	0
**Upper GI bleeding, n (%)**	1(0.9%)	0(0.0%)	1(0.2%)	0.230	-0.13
**Unpredictable ICU care, n (%)**	0(0.0%)	0(0.0%)	0(0.0%)	1.000	0.00
**Blood transfusion, n (%)**	15(13.5%)	40(10.8%)	55(11.4%)	0.422	-0.08

Data are presented as n (%). *P-value < 0.05 was considered statistically significant after test.

SMD: Standardized mean difference, NSAIDs: non-steroidal anti-inflammatory drugs, GI: gastrointestinal, ICU: intensive care unit.

Table S3. Primary and Secondary Outcomes During the Period of 2013-2018 (N=483)

	Control	Parecoxib	Total	P-value	SMD
N	111	372	483		
**Primary outcome**					
Time to mobilization (SD) in days	2.4 (1.4)	2.0 (0.9)	2.1 (1.0)	0.002*	-0.30
**Secondary outcomes**					
Mean LOS (SD) in days	6.8 (2.0)	6.5 (2.4)	6.6 (2.3)	0.167	-0.16
Postoperative VAS (SD)					
Day 1	3.5 (1.7)	3.2 (1.3)	3.3 (1.4)	0.082	-0.18
Day 2	2.9 (1.1)	2.8 (1.1)	2.8 (1.1)	0.895	-0.01
Day 3	2.7 (0.9)	2.6 (1.0)	2.6 (1.0)	0.375	-0.10
Mean dosage of morphine (SD) in mg					
Day 1	9.0 (5.4)	7.2 (4.8)	5.2 (3.2)	0.004*	-0.31
Days 1 to 2	13.8 (9.0)	9.0 (6.6)	10.2 (7.2)	<0.001*	-0.65
Days 1 to 3	14.4 (11.4)	9.0 (7.2)	10.8 (9.0)	<0.001*	-0.70
Postoperative nausea and vomiting, n (%)	14(12.6%)	47(12.6%)	61(12.6%)	0.995	0

Data are presented as mean (SD). *P-value < 0.05 was considered statistically significant after test.

SMD: Standardized mean difference, SD: standard deviation, VAS: visual analogue scale, LOS: length of stay.

Table S4. Variables Associated with Time to Mobilization (days) During the Period of 2013-2018 (N=483)

Predictor	Multivariate	
	β (95% CI)	P-value
**Group** (Parecoxib vs. Control)	-0.283 (-0.500, -0.066)	0.011*
**Age**	-0.013 (-0.025, -0.002)	0.021*
**Gender** (Male vs. Female)	0.245 (0.029, 0.461)	0.026*
**Tourniquet** (Yes vs. No)	0.340 (-0.126, 0.806)	0.153
**LIA** (Yes vs. No)	0.215 (-0.095, 0.524)	0.174
**Hypertension** (Yes vs. No)	0.004 (-0.195, 0.204)	0.965
**Diabetes mellitus** (Yes vs. No)	0.080 (-0.136, 0.295)	0.467
**ASA classification** (1~2 vs. ≧3)	-0.099 (-0.307, 0.109)	0.350
**OP time**	0.004 (0.001, 0.008)	0.024*

Dependent variable: postoperative time to mobilization. *P-value < 0.05 was considered statistically significant after test. LIA: local infiltration analgesia, ASA: American Society of Anesthesiologists, OP: operation, CI: confidence interval

Table S5. Variables Associated with Length of Stay (LOS) During the Period of 2013-2018 (N=483)

Predictor	Multivariate	
	**β (95% CI)**	**P-value**
**Group** (Parecoxib vs. Control)	-0.109 (-0.607, 0.39)	0.669
**Age**	-0.017 (-0.042, 0.008)	0.191
**Gender** (Male vs. Female)	0.435 (-0.056, 0.926)	0.082
**Tourniquet** (Yes vs. No)	0.123 (-0.950, 1.196)	0.822
**LIA** (Yes vs. No)	-0.008 (-0.713, 0.698)	0.983
**Hypertension** (Yes vs. No)	0.123 (-0.331, 0.576)	0.595
**Diabetes mellitus** (Yes vs. No)	0.319 (-0.176, 0.813)	0.206
**ASA classification** (1~2 vs. ≧3)	0.081 (-0.389, 0.550)	0.736
**OP time**	0.019 (0.010, 0.028)	<0.001*

Dependent variable: length of stay. *P-value < 0.05 was considered statistically significant after test. LIA: local infiltration analgesia, ASA: American Society of Anesthesiologists, OP: operation, CI: confidence interval
